# Applying both the 30-s and the 5-repetition sit-to-stand tests captures dissimilar groups and a broader spectrum of physical abilities in mobility-limited older individuals: results from the BIOFRAIL study

**DOI:** 10.1007/s41999-024-01115-6

**Published:** 2024-12-07

**Authors:** P. Hansen, H. Nygaard, J. Ryg, M. T. Kristensen, C. Suetta

**Affiliations:** 1 Geriatric Research Unit, Department of Medicine, Copenhagen University Hospital, Herlev and Gentofte, Gentofte, Denmark; 2 Geriatric Research Unit, Department of Geriatric and Palliative Medicine, Copenhagen University Hospital, Bispebjerg and Frederiksberg, Copenhagen, Denmark; 3 Faculty of Health and Medical Sciences, CopenAge, Copenhagen Center for Clinical Age Research, Institute of Clinical Medicine, University of Copenhagen, Copenhagen, Denmark; 4 Department of Emergency Medicine, Copenhagen University Hospital, Bispebjerg and Frederiksberg, Copenhagen, Denmark; 5 Department of Geriatric Medicine, Odense University Hospital, Odense, Denmark; 6 Geriatric Research Unit, Department of Clinical Research, University of Southern Denmark, Odense, Denmark; 7 Department of Physical and Occupational Therapy, Copenhagen University Hospital, Bispebjerg and Frederiksberg, Copenhagen, Denmark; 8 Faculty of Health and Medical Sciences, Institute of Clinical Medicine, University of Copenhagen, Copenhagen, Denmark

**Keywords:** Muscle strength, Muscle function, Lower limb, Mobility-limited patients, Old age

## Abstract

**Purpose:**

We aimed to assess differences among older patients demonstrating low STS performance in the 30 s-STS and/or the 5r-STS.

**Methods:**

30 s-STS and 5r-STS were used to assess lower limb muscle strength and function in older adults. Analysis involved 376 patients (≥ 65 years) from a geriatric outpatient clinic for fall assessment.

**Results:**

The mean age of patients was 79.8 (± 6.1) years (67% female). In total, 40.6% had low STS performance with 9.3% presenting only low 30 s-STS, 9.8% only low 5r-STS, and 21.5% low STS performance in both tests. Patients with low STS performance in both tests had lower gait speed, were more often frail, and had more prior falls compared to patients with low STS performance in one test only.

**Conclusion:**

The two STS tests are not interchangeable, and the use of both STS tests capture a wider range of physical abilities in mobility-limited older adults.

**Clinical trial registration:**

NCT05795556.

## Key summary points

**Aim:**

To evaluate differences among older patients demonstrating low sit-to-stand (STS) performance in the 30-s STS test (30 s-STS) and/or the 5-repetition STS test (5r-STS).

**Findings:**

Patients with low performance in both STS tests had lower gait speed, were more frail, and had more prior falls compared to patients with low performance in one test only.

**Message:**

The 30 s-STS and the 5r-STS test cannot be used interchangeable and represent different aspects of physical function.

## Background

Several factors have been demonstrated to increase the risk of falling, including muscle weakness, and in many cases, falling is a symptom of decline in functional status in older adults [1–3]. Moreover, muscle strength is crucial for older adults to perform daily activities, maintain proper posture and balance, and acts as primary indicator of sarcopenia [4]. Hence, lower body strength and functional mobility are key components in fall assessment in older adults.

The sit-to-stand (STS) test is commonly used to assess an individual’s ability to rise from a seated position, providing valuable insights into lower limb muscle strength, endurance, power, and functional mobility in older adults [5–8]. Different versions of the STS test exist, including the 30-s sit-to-stand test (30 s-STS) [6] and the 5-repetition sit-to-stand test (5r-STS) [5]. Whereas 30 s-STS evaluates the number of STS transitions performed in 30 s [6, 7], and the 5r-STS measures the time taken to complete five STS cycles [5]. Notably, the 5r-STS test is also a component of the Short Physical Performance Battery (SPPB), which is widely used to assess lower body function in older adults [5].

In Denmark, the 30 s-STS is commonly used as part of the initial assessment of older patients with frailty [9, 10]. According to the European Working Group on Sarcopenia in Older People 2 (EWGSOP2), both the 30 s-STS and the 5r-STS can be used to measure lower limb muscle strength [4]. Yet, it remains unclear whether the two tests identify low performance in the same patients and the same aspects of physical performance in older individuals.

The aim of this study was to assess potential differences related to demographic, clinical, and physiological characteristics among older patients demonstrating low STS performance in the 30 s-STS test and/or the 5r-STS test.

## Methods

### Design and settings

This study used data from the BIOFRAIL study, a cross-sectional cohort study including older patients (≥ 65 years) referred to the geriatric outpatient clinic for fall assessment at Copenhagen University Hospital, Herlev and Gentofte, Denmark, between September 2021 and June 2023 (Clinical Trial registration: NCT05795556). Exclusion criteria were: (i) known severe dementia, (ii) inability to provide informed consent or to adhere to the test protocol, and (iii) inability to walk independently without personal assistance (use of walking aids was permitted). The study was approved by the local Ethics Committee of Copenhagen (H-20057620) and was conducted in compliance with the Declaration of Helsinki. All patients provided written informed consent.

### Measurements

Patient characteristics included age, sex, and body mass index (BMI). STS performance was measured by the 30 s-STS test and the 5r-STS test [5, 6]. Patients were seated in the middle of a standardized chair, back straight, feet approximately a shoulder width apart, and arms across the chest. Verbal encouragement was given during the test. The patients were allowed to try both tests one time before the maximum trial. Low 30 s-STS performance was evaluated using the cut-off ≤ 8 repetitions [11] and low 5r-STS performance was defined as using more than 15 s for the 5 reps [4].

The 9-point Clinical Frailty Scale (CFS) was used to evaluate the presence of frailty defined by a score ≥ 5 [12]. Handgrip strength (HGS) was assessed using a hand-held dynamometer (Jamar Smart, Sammons Preston Rolyan, Chicago, Illinois, USA). A minimum of three attempts were made using the dominant hand, and the highest value obtained was selected for statistical analysis. Cut-off values for low HGS were < 27 kg for male and < 16 kg for female [4].

Appendicular lean mass (ALM) was assessed using direct segmental multi-frequency bioelectrical impedance analyses (DSM-BIA) (Inbody770 and InbodyS10; Biospace Co., Seoul, Korea) and reported as skeletal muscle index (SMI) (ALM (kg) adjusted for height^2^). Cut-off values for low muscle mass were < 7.0 kg/m^2^ and < 5.5 kg/m^2^, for male and female, respectively [4].

Gait speed (GS) was evaluated at maximal speed during horizontal walking over a 6-m course, with patients permitted to use their usual walking aids. The cut-off for low GS was ≤ 0.8 m/s [4]. Fall incidents within the past year were reported by the patient as part of the SARC-F questionnaire and classified as either no falls, 1–3 falls, or 4 or more falls [13].

### Statistical analysis

Descriptive statistics were applied and low 30 s-STS and low 5r-STS were reported as the relative frequency in percentage (%). Patients were grouped based on whether they presented low values in 30 s-STS test, 5r-STS test, or both tests. Potential differences between the groups with only low values on the 30 s-STS or the 5r-STS were investigated using independent t tests, non-parametric test, or Chi2, as appropriate. Corresponding statistics were applied to investigate potential differences between the group with low values on both STS tests and the group with low value on only one of the two tests. P-values ≤ 0.05 were considered statistically significant. Statistical analysis was performed in SAS Studio.

## Results

**Fig. 1:**
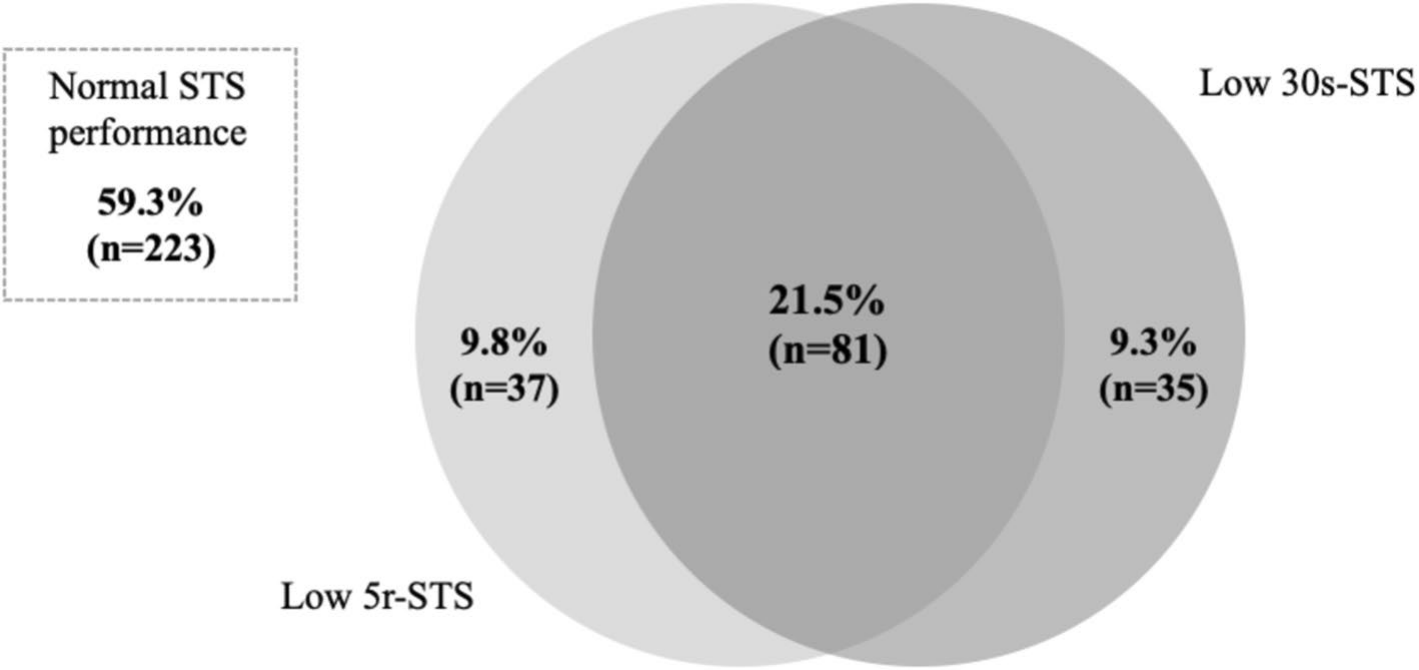
A proportional Venn diagram showing the overlap of low performance on sit-to-stand test (STS) between the two tests, the 30 s-STS and the 5r-STS, in older geriatric outpatients. A total of 376 patients, of whom 223 (59.4%) did not have low STS performance

This study included 376 patients (67% females (*n* = 252)), mean ± SD age 79.8 ± 6.1 years, and median [Q1;Q3] BMI 25.9 [23.0;28.4] kg/m^2^. Overall, 40.6% (n = 153) of the patients had low STS performance. Of these, 9.3% had only low 30 s-STS, 9.8% had only low 5r-STS, and 21.5% had low STS performance in both tests (Fig. 1).

No significant differences were seen in age, BMI, frailty, low HGS, muscle mass, GS, or the number of falls in the past year, between patient characteristics according to the presence of low performance in either the 30 s-STS or the 5r-STS.

When comparing the group of patients who demonstrated low STS performance in both tests with the patients who exhibited low STS performance in one test only, the group with low performance in both tests had a significantly higher proportion of frailty, lower GS, and experienced more prior falls (Table 1).

**Table 1:** Patient characteristics of the group who demonstrated low STS performance in both tests compared to the presence of either low 30 s-STS or 5r-STS performance

	All (*n* = 376)	Low STS both tests (*n* = 81, 21.5%)	Low 30 s-STS (*n* = 35, 9.3%)	*p*	Low 5r-STS (n = 37, 9.8%)	*p*
Age (years), Mean (SD)	79.8 (6.1)	80.9 (6.6)	80.9 (5.4)	0.18	80.4 (4.8)	0.64
Sex—female, *n* (%)	252 (67.0)	60 (74.1)	23 (65.7)	0.20	23 (62.2)	0.20
BMI (kg/m^2^), Median (Q1;Q3)	25.2 (23.0;28.4)	26.3 (22.6;29.0)	24.6 (22.7;28.1)	0.26	24.3 (22.4;27.2)	0.17
Frailty (CFS ≥ 5), *n* (%)	67 (19.4)	33 (45.2)	6 (18.2)	** < 0.01**	6 (17.7)	** < 0.01**
Low HGS^a^, *n* (%)	106 (28.6)	32 (40.5)	13 (37.1)	0.84	9 (25.0)	0.14
Male, *n* (%)	32 (26.2)	10 (50.0)	5 (41.7)		3 (21.4)	
Female, *n* (%)	74 (29.7)	22 (37.3)	8 (34.8)		6 (27.3)	
Low SMI^b^, *n* (%)	40 (11.3)	10 (13.0)	5 (16.7)^α^	0.76	3 (8.1)	0.54
Male, *n* (%)	10 (8.7)	1 (5.3)	1 (9.1)		0 (0.0)	
Female, *n* (%)	30 (12.6)	9 (15.5)	4 (21.1)		3 (13.0)	
Low GS, *n* (%)	43 (11.9)	24 (31.2)	5 (14.3)	0.07	2 (2.7)^β^	** < 0.01**
Self-reported falls during the past year, *n* (%)	67 (19.4)	68 (84.0)	23 (65.7)	** < 0.03**	27 (73.0)	0.13

Bold values indicate significant of *p*-values

*30s-STS* 30 s sit-to-stand, *5r-STS* 5 repetitions sit-to-stand, *BMI* Body Mass Index, *CFS* Clinical Frailty Scale, *GS* gait speed, *HGS* Handgrip strength, *SMI* skeletal muscle index, *STS* sit-to-stand

^a^*n* = 371 (male *n* = 122, female *n* = 249)

^b^*n* = 353 (male *n* = 115, female *n* = 238)

^α^*n* = 30, ^β^*n* = 35

## Discussion

The main finding of the study was the 30 s-STS and the 5r-STS test did not identifying low STS performance in the same patients, which may raise a discussion regarding the selection of which sit-to-stand tests to use in clinical practice among older adults. Our findings suggest that the 30 s-STS and the 5r-STS tests capture different aspects of lower body function and potentially identify different groups of patients at risk for falls, frailty, and functional decline.

In total, 40.6% (*n* = 153) of the patients had low STS performance. Of these, 21.5% (*n* = 81) had low performance in both STS tests, suggesting an ~ 50% overlap between the two tests in identifying individuals with lower limb muscle strength and functional impairments. Notably, patients with low performance in both tests had lower GS, higher prevalence of frailty, and experienced more falls compared to those with low performance in one test only, indicating these patients are at higher risk of adverse outcomes [14].

The finding that there is only ~ 50% overlap between the two STS tests highlights that the two STS tests capture different aspects of physical function. The 30 s-STS test measures a combination of muscle endurance and repetitive strength and is affected by motivation, balance, technique, and fatigue [6, 7]. In contrast, the 5r-STS test typically takes considerable shorter time, is more sensitive to lower limb muscle power, and less sensitive to changes in endurance [5, 8, 15]. Furthermore, the 5r-STS test tends to miss patients with very low muscle strength who cannot complete all five STS cycles [5, 8]. Therefore, the 30 s-STS is the most recommended and commonly used STS test in Denmark, whereas EWGSOP2 recommend that either of the 30 s-STS or the 5r-STS can be used to measure lower limb muscle strength [4].

We did not show significant differences between the group of patients with either low 30 s-STS or 5r-STS performance, which may be due to sample size. Future studies using larger samples should explore possible between group differences in other physiological parameters such as muscle power.

Our findings have important clinical implications for functional assessment in older adults. Using both the 30 s-STS and the 5r-STS tests in clinical and research settings will provide a more comprehensive assessment of older individual's physical capabilities. By doing so, practitioners can obtain a comprehensive evaluation of lower limb muscle strength, endurance, and power, thereby improving the accuracy of risk assessments and better fall prevention. However, we acknowledge that implementing both tests slightly extend the assessment time and therefore may not be possible in all settings. Moreover, some frail patients may have difficulty in performing both tests and the choice of test should, therefore, be setting dependent. Yet, it is noteworthy that these two tests that often are used interchangeable seems to represent different dimensions of physical function. Further research will help clarify the specific strengths and limitations of each test.

In conclusion, both the 30 s-STS and the 5r-STS are valuable for assessing lower limb muscle strength and functional performance in older adults. More importantly, the two STS assessments captures, to some extent, different aspects regarding physical ability and thereby risk of falls.

## Data Availability

The data used in the analysis for the present study are available from the corresponding author [PH] upon reasonable request.
